# Adversarial and Random Transformations for Robust Domain Adaptation and Generalization

**DOI:** 10.3390/s23115273

**Published:** 2023-06-01

**Authors:** Liang Xiao, Jiaolong Xu, Dawei Zhao, Erke Shang, Qi Zhu, Bin Dai

**Affiliations:** Unmanned Systems Technology Research Center, Defense Innovation Institute, Beijing 100071, China; xiaoliang@nudt.edu.cn (L.X.); jiaolong_xu@126.com (J.X.); adamzdw@163.com (D.Z.); bindai.cs@gmail.com (B.D.)

**Keywords:** image classification, domain adaptation, domain generalization, consistency training, spatial transformer networks, adversarial transformations

## Abstract

Data augmentation has been widely used to improve generalization in training deep neural networks. Recent works show that using worst-case transformations or adversarial augmentation strategies can significantly improve accuracy and robustness. However, due to the non-differentiable properties of image transformations, searching algorithms such as reinforcement learning or evolution strategy have to be applied, which are not computationally practical for large-scale problems. In this work, we show that by simply applying consistency training with random data augmentation, state-of-the-art results on domain adaptation (DA) and generalization (DG) can be obtained. To further improve the accuracy and robustness with adversarial examples, we propose a differentiable adversarial data augmentation method based on spatial transformer networks (STNs). The combined adversarial and random-transformation-based method outperforms the state-of-the-art on multiple DA and DG benchmark datasets. Furthermore, the proposed method shows desirable robustness to corruption, which is also validated on commonly used datasets.

## 1. Introduction

For modern computer vision applications, we expect a model trained on large-scale datasets to be able to perform uniformly well across various testing scenarios. For example, consider the perception system of a self-driving car; we want it to be able to generalize well across weather conditions and city environments. However, current supervised-learning-based models remain weak when it comes to out-of-distribution generalization [1]. When testing and training data are drawn from different distributions, the model can suffer from a significant accuracy drop. This is known as the domain shift problem, which has drawn increasing attention in recent years [1,2,3,4].

Domain adaptation (DA) and domain generalization (DG) are two typical techniques used to address the domain shift problem. DA and DG aim to utilize one or multiple labeled source domains to learn a model that performs well on an unlabeled target domain. The major difference between DA and DG is that DA methods require target data during training, whereas DG methods do not require target data in the training phase. DA can be categorized as supervised, semi-supervised, and unsupervised, depending on the availability of the labels of target data. In this paper, we consider unsupervised DA, which does not require labels of target data. In recent years, many works have been proposed to address either DA or DG problems [3,5]. In this work, we address both DA and DG in a unified framework.

Data augmentation is an effective technique for reducing overfitting and has been widely used in many computer vision tasks to improve the generalization ability [6] of the model. Recent studies show that using worst-case transformations or adversarial augmentation strategies can greatly improve the generalization and robustness of the model [7,8]. However, due to the non-differentiable properties of image transformations, searching algorithms such as reinforcement learning [9,10] or the evolution strategy [8] have to be applied, which are not computationally practical for large scale problems. In this work, we are concerned with the effectiveness of data augmentation for DA and DG, especially the adversarial data augmentation strategies, without using heavy-searching-based methods.

Motivated by the recent success of RandomAugment [11] on improving the generalization of deep learning models and consistency training in semi-supervised and unsupervised learning [12,13,14], we propose a unified DA and DG method by incorporating consistency training with random data augmentation. The idea is quite simple. When conducting a forward pass in neural networks, we force the randomly augmented and non-augmented pair of training examples to have similar responses by applying a consistency loss. Because consistency training does not require labeled examples, we can apply it with unlabeled target domain data for domain adaptation training. Consistency training and source-domain supervised training are both within a joint multi-task training framework and can be trained end-to-end. Random augmentation can also be regarded as a method for noisy injection, and by applying consistency training with noisy and original examples, the model’s generalization ability is expected to be improved. Following VAT [15] and UDA [13], we use KL divergence to compute the consistency loss.

To further improve the accuracy and robustness, we consider employing adversarial augmentations to find worst-case transformations. Our interest is in performing adversarial augmentation for DA and/or DG without using searching-based methods. Most image transformations are non-differentiable, except for a subset of geometric transformations. Inspired by the spatial transformer networks (STNs) of [16], we propose a differentiable adversarial spatial transformer network for both DA and DG. As we will show in the experimental section, the adversarial STN alone achieves promising results on both DA and DG tasks. When combined with random image transformations, it outperforms the state of the art, which is validated on several DA and DG benchmark datasets.

In this work, apart from the cross-domain generalization ability, robustness is also our concern. This is particularly important for real applications when applying a model to unseen domains, which, however, is largely ignored in current DA and DG literature. We evaluate the robustness of our models on **CIFAR-10-C** [17], which is a robustness benchmark with 15 types of corruptions algorithmically simulated to mimic real-world corruptions. The experimental results show that our proposed method not only reduces the cross-domain accuracy drop but also improves the robustness of the model.

Our contributions can be summarized as follows:

(1) We build a unified framework for domain adaptation and domain generalization based on data augmentation and consistency training.
(2) We propose an end-to-end differentiable adversarial data-augmentation strategy with spatial transformer networks to improve accuracy and robustness.
(3) We show that our proposed methods outperform state-of-the-art DA and DG methods on multiple object recognition datasets.
(4) We show that our model is robust to common corruptions and obtained promising results on the **CIFAR-10-C** robustness benchmark.

## 2. Related Work

### 2.1. Domain Adaptation

Modern domain adaption methods usually address domain shifts by learning domain-invariant features. This purpose can be achieved by minimizing a certain measure of domain variance, such as the Maximum Mean Discrepancy (MMD) [1,18] and fuzzy MMD [19], or aligning the second-order statistics of the source and target distributions [20,21].

Another line of work uses adversarial learning to learn features that are discriminative in source space and at the same time invariant with respect to domain shift [2,22,23]. In [2], a gradient reverse layer is proposed to achieve domain-adversarial learning by back-propagation. In [22], a method that combines adversarial learning and MMD is proposed. Ref. [23] outlined a generalized framework for adversarial adaptation and proposed ADDA, which uses an inverted label GAN loss to enforce domain confusion. In [24], a multi-layer adversarial DA method was proposed, in which a feature-level domain classifier is used to learn domain-invariant representation while a prediction-level domain classifier is used to reduce domain discrepancy in the decision layer. In [3], CycleGAN [25]-based unpaired image translation is employed to achieve both feature-level and pixel-level adaptation. In [26], cluster assumption is applied to domain adaptation, and a method called Virtual Adversarial Domain Adaptation (VADA) is proposed. VADA utilizes VAT [15] to enforce classifier consistency within the vicinity of samples. Drop to Adapt [27] also enforces the cluster assumption by leveraging adversarial dropout. In [28], adversarial learning and self-training are combined, in which an adversarial-learned confusion matrix is utilized to correct the pseudo label and then align the feature distribution.

Recently, self-supervised-learning-based domain adaptation was proposed [4]. Self-supervised DA integrates a pretext learning task, such as image rotation prediction in the target domain with the main task in the source domain. Self-supervised DA has shown the capability of learning domain-invariant feature representations [4,29]. In [30], label-consistent contrastive learning is proposed for source-free domain adaptation.

### 2.2. Domain Generalization

Similar to domain adaptation, existing work usually learns domain-invariant features by minimizing the discrepancy between the given multiple source domains, assuming that the source-domain-invariant feature works well for the unknown target domain. Domain-Invariant Component Analysis (DICA) is proposed in [31] to learn an invariant transformation by minimizing the dissimilarity across domains. In [32], a multi-domain reconstruction auto-encoder is proposed to learn domain-invariant features.

Adversarial learning has also been applied in DG. In [33], an MMD-based adversarial autoencoder (AAE) is proposed to align the distributions among different domains and match the aligned distribution to an arbitrary prior distribution. In [34], correlation alignment is combined with adversarial learning to minimize the domain discrepancy. In [35], optimal transport with Wasserstein distance is adopted in the adversarial learning framework to align the marginal feature distribution over all the source domains.

Some work utilizes the low-rank constraint to achieve domain generalization capability, such as [36,37,38]. Meta-learning has recently been applied to domain generalization, including [39,40,41]. In [42], a method integrated adversarial learning and meta-learning was proposed.

In [5,43], self-supervised DG is proposed by introducing a secondary task to solve a jigsaw puzzle and/or predict image rotation. This auxiliary task helps the network to learn the concepts of spatial correlation while acting as a regularizer for the main task. With this simple model, state-of-the-art domain generalization performance can be achieved.

### 2.3. Data Augmentation

Data augmentation is a widely used trick in training deep neural networks. In visual learning, early data augmentation usually uses a composition of elementary image transformation, including translation, flipping, rotation, stretching, shearing, and adding noise [44]. Recently, more complicated data augmentation approaches have been proposed, such as CutOut [45], Mixup [46], and AugMix [47]. These methods are designed by human experts based on prior knowledge of the task, together with trial and error. To automatically find the best data augmentation method for a specific task, policy-search-based automated data-augmentation approaches have been proposed, such as AutoAugment [9] and Population based augmentation (PBA) [48]. The main drawback of these automated data augmentation approaches is the prohibitively high computational cost. Recently, Ref. [7] improved the computational efficiency of AutoAugment by simultaneously optimizing target-related object and augmentation policy search loss.

Another kind of data augmentation method aims at finding the worst-case transformations and utilizing them to improve the robustness of the learned model. In [49], adversarial data augmentation is employed to generate adversarial examples, which are appended during training to improve the generalization ability. In [8], the authors further proposed searching for worst-case image transformations by random search or evolution-based search. Reinforcement learning is used in [7] to search for adversarial examples, in which RandAugment and worst-case transformation are combined.

Recently, consistency training with data augmentation has been used for improving semi-supervised training [13] and the generalization ability of supervised training [50].

Most related works focus on either domain adaptation or domain generalization, while in this work, we consider designing a general model to address both of them. Domain adversarial training is a widely used technique for DA and DG, and our work does not follow this mainstream methodology but seeks resolution from the perspective of representation learning, e.g., self-supervised learning [4], and consistency learning [29]. For representation learning, data augmentation also plays an important role, as it can reduce model overfitting and improve the generalization ability. However, whether data augmentation can address cross-domain adaptation and generalization problems is still not well explored. In this work, we design a framework to incorporate data augmentation and consistency learning to address both domain adaptation and generalization problems.

## 3. The Proposed Approach

In this section, we present the proposed method for domain adaptation and generalization in detail.

### 3.1. Problem Statement

In the domain adaptation and generalization problem, we are given a source domain $D_{s}$ and target domain $D_{t}$ containing samples from two different distributions, $P_{S}$ and $P_{T}$. Denoting by $\left\{x^{s},{\hat{y}}^{s}\right\}\in D_{s}$ a labeled source domain sample and by $\left\{x^{t}\right\}\in D_{t}$ a target domain sample without label, we have $x^{s}∼P_{S}$, $x^{t}∼P_{T}$, and $P_{S}\neq P_{T}$. When applying the model trained on the source domain to the target domain, the distribution mismatch can lead to a significant performance drop.

The task of unsupervised domain adaptation is to train a classification model $F:x^{s}\to y^{s}$ that is able to classify $x^{t}$ to the corresponding label $y^{t}$ given $\left\{x^{s},{\hat{y}}^{s}\right\}$ and $\left\{x^{t}\right\}$ as training data. On the other hand, the task of domain generalization is to train a classification model $F:x^{s}\to y^{s}$ which is able to classify $x^{t}$ to the corresponding label $y^{t}$ given only $\left\{x^{s},{\hat{y}}^{s}\right\}$. The difference between these two tasks concerns whether $\left\{x^{t}\right\}$ is involved or not during training. For both domain adaptation and generalization, we assume there are $n_{s}$ source domains where $n_{s}⩾1$ and there is one single target domain.

Many works have addressed either domain adaptation or domain generalization. In this work, we propose a unified framework to address both problems. In what follows, we first focus on domain adaptation and introduce the main idea and explain the details of the proposed method. Then, we show how this method can be adapted to domain generalization tasks as well.

### 3.2. Random Image Transformation with Consistency Training

**Figure 1:**
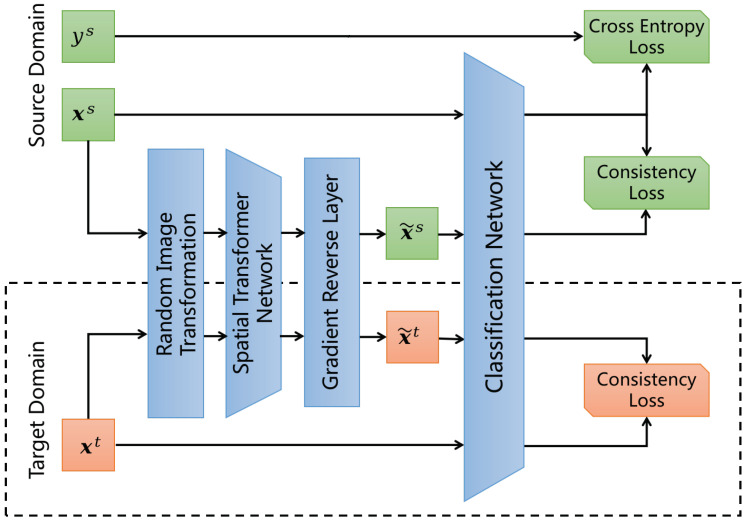
Overview of our proposed model. We propose using random image transformations and adversarial spatial transformer networks (STN) to achieve domain adaptation and generalization (without the dashed line bounding box).

Inspired by a recent line of work [13,29] in semi-supervised learning that incorporates consistency training with unlabeled examples to enforce the smoothness of the model, we propose using image transformation as a method for noisy injection and apply consistency training with the noisy and original examples. The overview of the proposed random image transformation with consistency training for domain adaptation is depicted in  Figure 1. In this section, we focus on the random image transformation part and leave the adversarial spatial transformer networks in the next section. The main idea can be explained as follows:

(1) Given an input image x from either the source or target domain, we compute the output distribution p(y∣x) with x and a noisy version $p(y|\tilde{x})$ by applying random image transformation to x;
(2) For domain adaptation, we jointly minimize the classification loss with labeled source-domain samples and a divergence metric between the two distributions $D(p\left(y|x\right)\|p\left(y|\tilde{x}\right))$ with unlabeled source and target domain samples, where D is a discrepancy measure between two distributions;
(3) For domain generalization, the procedure is similar to (2) but without using any target domain samples.

Our intuition is that, on one hand, minimizing the consistency loss can enforce the model to be insensitive to the noise and improve the generalization ability; on the other hand, the consistency training gradually transmits label information from labeled source domain examples to unlabeled target domain ones, which improves the domain adaptation ability.

The applied random-image transformations are similar to RandAugment [11]. Table 1 shows the types of image transformations used in this work. The image transformations are categorized into three groups. The first group is the geometric transformations, including Shear, Translation, Rotation, and Flip. The second group is the color-enhancing-based image transformations, e.g., Solarize, Contrast, etc., and the last group includes other transformations, e.g., CutOut and SamplePairing. Each type of image transformation has a corresponding magnitude, which indicates the strength of the transformation. The magnitude can be either a continuous or discrete variable. Following [11], we also normalize the magnitude to a range from 0 to 10, in order to employ a linear scale of magnitude for each type of transformation. In other words, a value of 10 indicates the maximum scale for a given transformation, while 0 means the minimum scale. Note that these image transformations are commonly used as searching policies in recent auto-augmentation literature, such as [7,9,10]. Following [11], we do not use search but instead uniformly sample from the same set of image transformations. Specifically, for each training sample, we uniformly sample $N_{aug}$ image transformations from Table 1 with the normalized magnitude value of $M_{aug}$ and then apply them to the image sequentially. $N_{aug}$ and $M_{aug}$ are hyper-parameters. Following the practice of [11], we sampled $N_{aug}\in \left\{1,2,3,5,10\right\}$ and $M_{aug}\in \left\{3,6,9,12\right\}$. We conducted validation experiments on the **PACS** dataset and **VisDA** dataset and found that $N_{aug}=2$ and $M_{aug}=9$ obtain the best results; thus, we keep $N_{aug}=2$ and $M_{aug}=9$ in all our experiments.

Following VAT [15] and UDA [13], we also use the KL divergence to compute the consistency loss. We denote by $\theta_{m}$ the parameters of the classification model. The classification loss with labeled source-domain samples is written as the following cross-entropy loss:

(1) $$L_{m}\left(\theta_{m}\right)=E_{x^{s},{\hat{y}}^{s}\in D_{s}}\left[-\log p({\hat{y}}^{s}|x^{s})\right].$$

The consistency loss for domain adaptation can be written as

(2) $$L_{c}\left(\theta_{m}\right)=E_{x\in D_{s}\cup D_{t}}E_{\tilde{x}\in \tilde{D_{s}}\cup \tilde{D_{t}}}\left[D_{\mathrm{KL}}\left(\hat{p}\left(y|x\right),p\left(y|\tilde{x}\right)\right)\right],$$

where $\hat{p}\left(y|x\right)$ uses a fixed copy of $\theta_{m}$, which means that the gradient is not propagated through $\hat{p}\left(y|x\right)$.

As a common underlying assumption in many semi-supervised learning methods, the classifier’s decision boundary should not pass through high-density regions of the marginal data distribution [51]. The conditional entropy minimization loss (EntMin) [52] enforces this by encouraging the classifier to output low-entropy predictions on unlabeled data. EntMin is also combined with VAT in [15] to obtain stronger results. Specifically, the conditional entropy minimization loss is written as

(3) $$L_{e}\left(\theta_{m}\right)=E_{x^{t}\in D_{t}}\left[-p\left(y^{t}|x^{t}\right)\log p(y^{t}|x^{t})\right].$$

Following [4,5], we also apply the conditional entropy minimization loss to the unlabeled target domain data to minimize the classifier prediction uncertainty. The full objective of domain adaptation is thus written as follows:

(4) $$J_{DA}\left(\theta_{m}\right)=\min_{\theta_{m}}\left(L_{m}+\lambda_{c}L_{c}+\lambda_{e}L_{e}\right),$$

where $\lambda_{c}$ and $\lambda_{e}$ are the weight factor for the consistency loss and conditional entropy minimization loss.

For domain generalization, as no target domain data are involved during the training, Equation (2) can be written as:

(5) $$L_{c}\left(\theta_{m}\right)=E_{x\in D_{s}}E_{\tilde{x}\in \tilde{D_{s}}}\left[D_{\mathrm{KL}}\left(\hat{p}\left(y|x\right),p\left(y|{\tilde{x}}^{t}\right)\right)\right],$$

and the final objective function is the weighted sum of Equation (5) and the classification loss Equation (1):

(6) $$J_{DG}\left(\theta_{m}\right)=\min_{\theta_{m}}\left(L_{m}+\lambda_{c}L_{c}\right).$$

### 3.3. Adversarial Spatial Transformer Networks

The proposed random image transformation with consistency training is a simple and effective method to reduce domain shift. Recent works show that using worst-case transformations or adversarial augmentation strategies can significantly improve the accuracy and robustness of the model [7,8]. However, most of the image transformations in Section 3.2 are non-differentiable, making it difficult to apply gradient-descent-based methods to obtain optimal transformations. To address this problem, searching algorithms such as reinforcement learning [7] or evolution strategy [8] have been employed in recent works, which, however, are computationally expensive and do not guarantee obtaining the global optima. In this work, we find that a subset of the image transformations in Table 1 are actually differentiable, i.e., the geometric transformations. In this work, we build our adversarial geometric transformation on top of the spatial transformer networks (STN) [16]. Specifically, in this work, we focus on the affine transformations. The STN consists of a localization network, a grid generator, and a differentiable image sampler. The localization network is a convolutional neural network with parameter $\theta_{t}$, which takes as input an image x and regresses the affine transformation parameters ϕ. The grid generator takes as input ϕ and generates the transformed pixel coordinates as follows:

(7) $$\left[\begin{array}{c} u\\ v \end{array}\right]=\phi \left[\begin{array}{c} \tilde{u}\\ \tilde{v}\\ 1 \end{array}\right]=\left[\begin{array}{ccc} \phi_{11} & \phi_{12} & \phi_{13}\\ \phi_{21} & \phi_{22} & \phi_{23} \end{array}\right]\left[\begin{array}{c} \tilde{u}\\ \tilde{v}\\ 1 \end{array}\right],$$

where $\left(\tilde{u},\tilde{v}\right)$ are the normalized transformed coordinates in the output image and (u,v) are the normalized source coordinates in the input image, i.e., $-1\leq \tilde{u},\tilde{v},u,v\leq 1$. Finally, the differentiable image sampler takes the set of sampling points from the grid generator, along with the input image x, and produces the sampled output image $\tilde{x}$. The bilinear interpolation is used during the sampling process. We can denote STN by $T:x\to \tilde{x}$, a differentiable neural network with parameter $\theta_{t}$, which applies an affine transformation to the input image x.

The goal of the adversarial geometric transformation is to find the worst-case transformations, which is equivalent to maximizing the following objective function:

(8) $$\underset{\theta_{t}}{\mathrm{argmax}}E_{x\in D_{s}\cup D_{t}}[D_{\mathrm{KL}}\left(\hat{p}\left(y|x\right),p\left(y|T\left(x\right)\right)\right].$$

The straightforward way to solve the maximization problem in Equation (8) is to apply the gradient reverse trick, i.e., the gradient reversal layer (GRL) in [2], which is popular in domain adversarial training methods. The GRL has no parameters associated with it. During the forward propagation, it acts as an identity transformation. During the back-propagation, however, the GRL takes the gradient from the subsequent level and changes its sign, i.e., multiplies it by −1, before passing it to the preceding layer. Formally, the forward and back propagation of GRL can be written as $R\left(x\right)=x,\frac{dR}{dx}=-I$. The loss function of the adversarial spatial transformer for domain adaptation can thus be written by

(9) $$L_{adv}\left(\theta_{t}\right)=E_{x\in D_{s}\cup D_{t}}[D_{\mathrm{KL}}\left(\hat{p}\left(y|x\right),p\left(y|R\left(T\left(x\right)\right)\right)\right],$$

where T is the spatial transformer network with parameter $\theta_{t}$, and R is the gradient reverse layer (GRL) [1]. For domain generalization, the only difference is that only $x\in D_{s}$ is involved in Equation (9). With the adversarial spatial transformer network, the final objective function for domain adaptation is written as

(10) $$\begin{array}{c} J_{DA}\left(\theta_{m},\theta_{t}\right)=\min_{\theta_{m}}\max_{\theta_{t}}\left(L_{m}+\lambda_{c}L_{c}+\lambda_{e}L_{e}+\lambda_{t}L_{adv}\right), \end{array}$$

and the final objective function for domain generalization is written as

(11) $$\begin{array}{c} J_{DG}\left(\theta_{m},\theta_{t}\right)=\min_{\theta_{m}}\max_{\theta_{t}}\left(L_{m}+\lambda_{c}L_{c}+\lambda_{t}L_{adv}\right). \end{array}$$

## 4. Experiments

In this section, we conduct experiments to evaluate the proposed method and compare the results with the state-of-the-art domain adaptation and generalization methods.

### 4.1. Datasets

Our method was evaluated on the following popular domain adaptation and generalization datasets:

**PACS** [53] is a standard dataset for DG. It contains 9991 images collected from Sketchy, Caltech256, TU-Berlin, and Google Images. It has 4 domains (Photo, Art Paintings, Cartoon, and Sketches), and each domain consists of 7 object categories. Following [5], we evaluated both domain generalization and multi-source domain adaptation on this dataset.

**ImageCLEF-DA** [54] is a benchmark dataset in the domain adaptation community for the ImageCLEF 2014 domain adaptation challenge. It consists of three domains, including Caltech-256 (C), ImageNet ILSVRC 2012 (I), and Pascal VOC 2012 (P). Each domain consists of 12 common classes. Six domain adaptation tasks are evaluated on ImageCLEF: I→P, P→I, I→C, C→I, C→P and P→C. There are 600 images in each domain and 50 images in each category.

**Office-Home** [55] was used for evaluating both domain adaptation and generalization. It contains 4 domains, and each domain consists of images from 65 categories of everyday objects. The 4 domains are Art (**A**), Clipart (**C**), Product (**P**), and Real-World (**R**). The Clipart domain is formed with clipart images. The Art domain consists of artistic images in the form of paintings, sketches, ornamentation, etc. The Real-World domain’s images are captured by a regular camera and the Product domain’s images have no background. The total number of images is about 15,500.

**VLCS** [56] was used for evaluating domain generalization. It contains images of 5 object categories shared by 4 separate domains: PASCAL VOC 2007, LabelMe, Caltech and Sun datasets. Unlike Office-Home and PACS, which are related in terms of domain types, VLCS offers different challenges because it combines object categories from Caltech with scene images of the other domains.

**VisDA** http://ai.bu.edu/visda-2017/ (accessed on 10 May 2020) is a simulation-to-real domain-adaptation dataset that has over 280 K images across 12 classes. The synthetic domain contains renderings of 3D models from different angles and with different lighting conditions, and the real domain contains nature images.

To investigate the robustness of the proposed model, we also evaluated it on popular robustness benchmarks, including the following.

**CIFAR-10.1** [57] is a new test set of **CIFAR-10** with 2000 images and the exact same classes and image dimensionality. Its creation follows the creation process of the original **CIFAR-10** paper as closely as possible. The purpose of this dataset is to investigate the distribution shifts present between the two test sets, and the effect on object recognition.

**CIFAR-10-C** [17] is a robustness benchmark where 15 types of corruption are algorithmically simulated to mimic real-world corruption as much as possible on copies of the **CIFAR-10** [58] test set. The 15 types of corruption are from four broad categories: noise, blur, weather, and digital. Each corruption type comes in five levels of severity, with level 5 being the most severe. In this work, we evaluated the models with the level 5 severity.

### 4.2. Experimental Setting

We implemented the proposed method using the PyTorch framework on a single RTX 2080 Ti GPU with 11 GB memory. The Alexnet [59], Resnet-18, and Resnet-50 [60] architectures were used as base networks and initialized with ImageNet [61] pretrained weights.

For training the model, we used an SGD solver with an initial learning rate of 0.001. We trained the model for 60 epochs and decayed the learning rate to 0.0001 after 80% of the training epochs. For training baseline models, we used simple data-augmentation protocols by random cropping, horizontal flipping, and color jittering.

We followed the standard protocol for unsupervised domain adaptation [2,62], where all labeled source domain examples and all unlabeled target domain examples were used for adaptation tasks. We also followed the standard protocol for domain-generalization transfer tasks as per [5], where the target domain examples were unavailable in the training phase. We set three different random seeds and ran each experiment three times. The final result is the average over the three repetitions.

We compared our proposed method with state-of-the-art DA and DG methods. The descriptions of the compared methods are shown in Table 2. In the following, we use *Deep All* to denote the baseline model trained with all available source-domain examples when all the introduced domain adaptive conditions are disabled. For the compared methods in Table 2, we used the results reported from the original papers if the protocol is the same.

### 4.3. Experimental Results

#### 4.3.1. Unsupervised Domain Adaptation

The multi-source domain adaptation-results on **PACS** are reported in Table 3. We follow the settings in [4,5] and trained our model considering three domains as the source datasets and the remaining one as the target. *RotC* is an improved version of *Rot*, which applies consistency loss with the simplest image rotation transformations [29]. We use the open source code of [65] to produce the results of *CDAN* and *CDAN+E* and the open source code of [66] to produce the results of *MDD*. Our proposed approach outperforms all baseline methods on all transfer tasks. The last column shows the average accuracy on the four tasks. Our proposed approach outperforms state-of-the-art *CDAN+E* [65] by 4.7 percentage points and *MDD* by 1.4 percentage points.

To investigate the improvement from data augmentation, we add the same type of data augmentation as ours on *Deep All*, *DANN*, *CDAN*, *CDAN+E*, and *MDD*, which are denoted by *Deep All (Aug)*, *DANN (Aug)*, *CDAN (Aug)*, *CDAN+E (Aug)*, and *MDD (Aug)*, respectively. From these results, we can see that data augmentation can obtain an improvement of 1.2 to 2.6 percentage points for existing domain-adaptation methods. Even with the same type of data augmentation, our proposed method still outperforms these baselines.

The results on **Office-Home** dataset are reported in Table 4. On the **Office-Home** dataset, we conducted 12 transfer tasks of four domains in the context of single-source domain adaptation. We achieved state-of-the-art performance on 8 out of 12 transfer tasks. It is noted that although **Office-Home** and **PACS** are related in terms of domain types, the number of total categories in **Office-Home** and **PACS** are 65 and 7, respectively. From the results, we can see that the proposed method scales when the number of categories changes from 7 to 65. The average accuracy achieved by our proposed method is 67.6%, which outperforms all the compared methods.

The results on the **ImageCLEF-DA** dataset are reported in Table 5. As the three domains in **ImageCLEF-DA** are of equal size and balanced in each category and are visually more similar, there is little room for improvement in this dataset. Even so, our method still outperforms the comparison methods on four out of six transfer tasks. Our method achieves 88.2% average accuracy, outperforming the latest methods, including *CDAN+E* [65], *RotC* [29] and *MLADA* [24].

The proposed method also obtains strong results on **VisDA** as reported in Table 6. It outperforms *CDAN+E* by 2.6 percentage points.

It is important to understand the improvement of the consistency loss. Because *RotC* is the combination of simple image rotation transformation and consistency loss, without using complex data augmentation, we can better understand how much of the improvement is from the consistency loss. Comparing to *Rot*, which does not use consistency loss, we can see from the above DA experiments that *RotC* obtains an improvemnt of about 0.7 to 3.5 percentage points thanks to the consistency loss.

#### 4.3.2. Domain Generalization

In the context of multi-source domain generalization, we conducted four transfer tasks on **the PACS** dataset. We compared the performance of our proposed method against several recent domain-generalization methods. We evaluated the method with both Alexnet and Resnet-18 and report the results in Table 7 and Table 8. From the results, we can observe that our proposed method achieves state-of-the-art domain generalization performance with both backbone architectures. With Alexnet, our method outperforms the comparison methods on all 4 transfer tasks. The average accuracy of our method outperforms the prior best method *WADG* by around 1.7 percentage points, setting a new state-of-the-art performance. With Resnet-18, the average accuracy of our method is 82.73%, also outperforming the existing latest ones.

As consistency loss is not mandatory for DG, we replaced consistency loss with cross-entropy loss and ran our methods. The results are denoted by **Ours w/o consis**. We can see that the model trained with cross-entropy loss obtains similar accuracy to ours with consistency loss. However, the consistency loss is required for DA because of the unlabeled target domain samples. To keep a unified framework for both DA and DG problems, we used consistency loss for DG in this work.

To investigate the improvement of pure data augmentation without consistency, we ran *JiGen* and *Deep All* with the same type of data augmentation as ours, denoted by *JiGen (Aug)* and *Deep All (Aug)*. We can see that using pure augmentation, *Deep All* can obtain an improvement of around 0.8 to 1.2 percentage points, and *JiGen* can obtain an improvement of around 1.2 to 1.6 percentage points. Even so, our proposed method still outperforms these baselines.

We also conducted experiments on **Office-Home** and **VLCS** datasets for multi-source domain generalization. Compared to **PACS**, these two datasets are more difficult, and most recent works have only obtained small accuracy gains with respect to the *Deep All* baselines. The results on **Office-Home** and **VLCS** dataset are reported in Table 9 and Table 10, respectively. Our proposed method outperforms the compared methods on the four transfer tasks on **the Office-Home** dataset, and the results tested on the **VLCS** dataset show that our method achieves the best or close to the best performance on the four tasks, outperforming the recently proposed ones on average. It is noted that our baseline *Deep All* has relatively higher accuracy than other baselines. This is because we also add data augmentations such as random crop, horizontal flipping, and color jittering when training *Deep All* models. In this case, it is fairer to compare with the proposed method, which incorporates various data augmentation operations.

#### 4.3.3. Robustness

**Figure 2:**
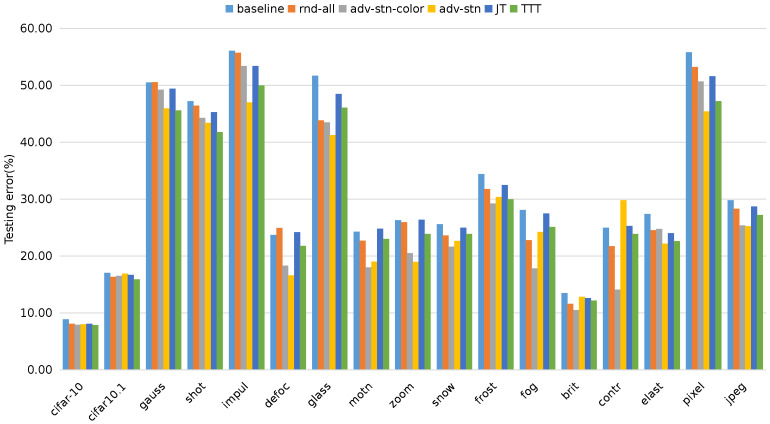
Test error (%) on CIFAR-10, CIFAR10.1 and CIFAR-10-C (level 5). Best viewed in color.

Apart from domain adaptation and generalization, we are also interested in the robustness of the learned model. In this part, we evaluate the proposed method on robustness benchmarks **CIFAR-10.1** and **CIFAR-10-C**. We trained on the standard **CIFAR-10** training set and tested on various corruption datasets, i.e., in a single-source domain-generalization setting. Figure 2 shows the testing error on different datasets. We evaluated different image transform strategies and also compare them with recently proposed methods in [74], i.e., *JT* and *TTT*. Following [74], we used the same architecture and hyper-parameters across all experiments.

The method denoted by *baseline* refers to the plain Resnet model, which is equivalent to *Deep All* in the DG setting. *JT* and *TTT* are the joint training and testing time training in [74], respectively. We denote by *rnd-all* the random image transformation including geometric and color-based transformations, *adv-stn* the proposed adversarial STN without random image transforms, and *adv-stn-color* the adversarial STN combined with random color-based transformations.

On the left is the standard **CIFAR-10** testing dataset, where we can see that all the compared methods obtained similar accuracies. On **CIFAR10.1**, the testing errors of all these methods increase simultaneously, but there is no significant gap between them. On the **CIFAR-10-C** corruption data sets, the performances of these methods vary a lot. Our proposed methods show improved accuracies compared to the *baseline*. *adv-stn-color* shows better performance than its variants and also outperforms *JT* and *TTT*. It can also be seen that *adv-stn* even outperforms *rnd-all* in most cases, although it only applies geometric transformations, which indicates the effectiveness of the proposed adversarial spatial transformations for improving robustness.

### 4.4. Ablation Studies and Analysis

Below, we focus on the PACS DG and DA setting for ablation analyses of the proposed method.

#### 4.4.1. Ablation Study on Image Transformation Strategies

In this part, we conducted ablation studies on adversarial and random image transformations. Table 11 shows the ablation studies of domain adaptation on PACS with different image transformation strategies, and Table 12 shows the domain-generalization results. *rnd-color* and *rnd-geo* are subsets of *rnd-all*, where *rnd-color* refers to color-based transformations and *rnd-geo* refers to geometric transformations. Please see Table 1 for details of each subset of transformations. For the DA task, when comparing individual transformation strategies, *rnd-color* obtained the best accuracy, and *adv-stn* outperformed *rnd-geo*. The combination of *rnd-color* + *adv-stn* also outperformed *rnd-color* + *rnd-geo*. However, in this experiment, *adv-stn* did not further improve *rnd-color*, which might be due to the limited room for improvement in the baseline method.

In the DG experiment, we obtained the similar conclusion that *adv-stn* outperforms *rnd-geo*. As the baseline accuracy of DG is far from saturated compared to DA, we can see that *adv-stn* further improved *rnd-color* and the combination of *rnd-color* + *adv-stn* obtained the best accuracy.

#### 4.4.2. Ablation Study on the Hyperparameters Setting

**Figure 3:**
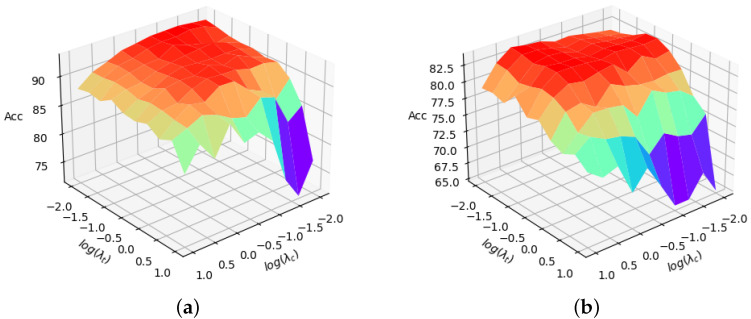
Accuracies with different hyperparameter settings on PACS **photo**, **cartoon**, **sketch**→**art_painting** task (Resnet-18). (**a**) Multi-source Domain adaptation with fixed $\lambda_{e}$. (**b**) Domain generalization. Best viewed in color.

In this part, we conducted ablation studies on the hyperparameters setting. The final objective functions of our proposed method for domain adaptation (10) and domain generalization (11) are a weighted summation of several items, with the weighting factors as the hyperparameters. Since the conditional entropy minimization loss is widely used in domain adaptation, we fixed the weight $\lambda_{e}=0.1$ and conducted a grid test with different settings of $\lambda_{c}$ and $\lambda_{t}$, which are shared by domain generalization. We tested ten different values, which are logarithmically spaced between $10^{-2}$ and 10 for $\lambda_{c}$ and $\lambda_{t}$. For each setting, we ran with three different random seeds and calculated the mean accuracy. The results of multi-source domain adaptation and domain generalization, which take **photo**, **cartoon**, and **sketch** as the source domains and **art_painting** as the target domain, are reported in Figure 3. Resnet-18 was used as the base network.

From the figures, we can see that when both $\lambda_{c}$ and $\lambda_{t}$ are not too large, the accuracies are relatively stable, validating the insensitiveness of our proposed method to hyperparameters. However, when $\lambda_{c}$ and $\lambda_{t}$ grow too large, the performance decreases, especially with a small $\lambda_{c}$ and a large $\lambda_{t}$. The reason may be that overwhelmingly large weights for consistency loss and adversarial spatial transformer loss over the main classification loss make the learned feature less discriminative for the main classification task, therefore resulting in lower accuracy. Moreover, too large $\lambda_{t}$ may lead to excessive emphasis on the extreme geometric distortions, which may be harmful to the general cross-domain performance.

#### 4.4.3. Visualization of Learned Deep Features

**Figure 4:**
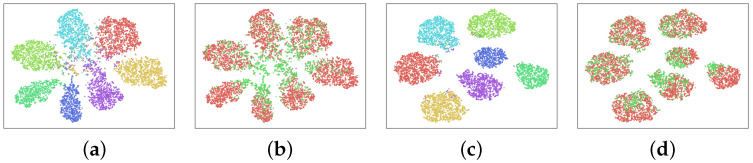
The t-SNE visualization on the PACS DA setting: (**a**) class visualization of *Deep All*, (**b**) domain visualization of *Deep All*, (**c**) class visualization of *Ours*, (**d**) domain visualization of *Ours*.

**Figure 5:**
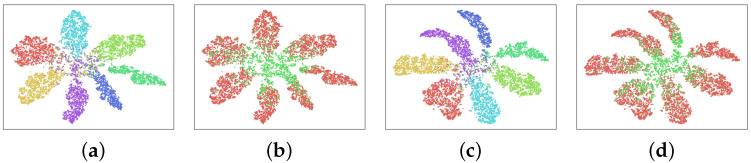
The t-SNE visualization on the PACS DG setting: (**a**) class visualization of *Deep All*, (**b**) domain visualization of *Deep All*, (**c**) class visualization of *Ours*, (**d**) domain visualization of *Ours*.

To better understand the learned domain-invariant feature representations, we use t-SNE [75] to conduct an embedding visualization. We conducted experiments on the transfer task of photo,cartoon,sketch→art_painting with both DA and DG settings and visualized the feature embeddings. Figure 4 shows the visualization on the PACS DA setting, and Figure 5 shows the visualization on the PACS DG setting. In both figures, we visualize category alignment as well as domain alignment. We also compare to the baseline *Deep All*, which does not apply any adaptation.

From the visualization of the embeddings, we can see that the clusters created by our model not only separate the categories but also mix the domains. The visualization from the DG model suggests that our proposed method is able to learn feature representations generalizable to unseen domains. It also implies that the proposed method can effectively learn domain-invariant representation with unlabeled target domain examples.

#### 4.4.4. Visualization of Adversarial Examples

**Figure 6:**
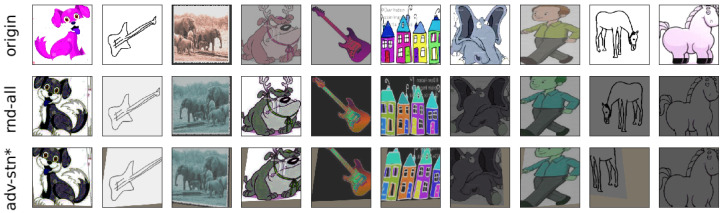
Visualization of the transformed images during the PACS DG training. First row: original image with random horizontal flipping and jittering; second row: original image with the proposed random augmentation; third row: the original image with the proposed adversarial spatial transformation combined with random color-based transformations.

To visually examine what the adversarial spatial transformations learned by *adv-stn* are, we plot the transformed examples during training in Figure 6. In the first row, we show the original images with simple random horizontal flipping and jittering augmentation. The second and third rows show images with *rnd-all* and *adv-stn-color*, respectively. From the figure, we can see that the proposed adversarial STN does find more difficult image transformations than random augmentation. Training with these adversarial examples greatly improves the generalization ability and robustness of the model.

## 5. Conclusions

In this work, we proposed a unified framework for addressing both domain adaptation and generalization problems. Our domain adaptation and generalization methods are built upon random image transformation and consistency training. This simple strategy can be used to obtain promising DA and DG performance on multiple benchmarks. To further improve its performance, we proposed a novel adversarial spatial transformer network that is differentiable and able to find the worst-case image transformation to improve the generalizability and robustness of the model. Experimental results on multiple object recognition DA and DG benchmarks verified the effectiveness of the proposed methods. Additional experiments tested on CIFAR-10.1 and CIFAR-10-C also validated the robustness of the proposed method.

## Figures and Tables

**Table 1:** Image transform operations. Some operations have discrete magnitude parameters, while others have no or continuous magnitude parameters.

	Name	Magnitude Type	Magnitude Range
Geometric transformations	ShearX	continuous	[0, 0.3]
	ShearY	continuous	[0, 0.3]
	TranslateX	continuous	[0, 100]
	TranslateY	continuous	[0, 100]
	Rotate	continuous	[0, 30]
	Flip	none	none
Color-based transformations	Solarize	discrete	[0, 255]
	Posterize	discrete	[0, 4]
	Invert	none	none
	Contrast	continuous	[0.1, 1.9]
	Color	continuous	[0.1, 1.9]
	Brightness	continuous	[0.1, 1.9]
	Sharpness	continuous	[0.1, 1.9]
	AutoContrast	none	none
	Equalize	none	none
Other transformations	CutOut	discrete	[0, 40]
	SamplePairing	continuous	[0, 0.4]

**Table 2:** The compared state-of-the-art methods on domain adaptation (DA) and domain generalization (DG) tasks. The column **Year** shows the year the method was published.

Methods	Task	Year	Description
**DANN** [2]	DA	2015	Domain adversarial training.
**DAN** [1]	DA	2015	Deep adaptation network.
**ADDA** [23]	DA	2017	Adversarial discriminative domain adaptation.
**JAN** [62]	DA	2017	Joint adaptation network.
**Dial** [63]	DA	2017	Domain alignment layers.
**DDiscovery** [64]	DA	2018	Domain discovery.
**CDAN** [65]	DA	2018	Conditional domain adversarial training.
**MDD** [66]	DA	2019	Adversarial training with margin disparity discrepancy.
**Rot** [4]	DA	2019	Self-supervised learning by rotation prediction.
**RotC** [29]	DA	2020	Self-supervised learning with consistency training.
**ALDA** [28]	DA	2020	Adversarial-learned loss for domain adaptation.
**MLADA** [24]	DA	2021	Multi-layer adversarial domain adaptation.
**GPDA** [67]	DA	2021	Geometrical preservation and distribution alignment.
**CCSA** [68]	DA and DG	2017	Embedding subspace learning.
**JiGen** [5]	DA and DG	2019	Self-supervised learning by solving jigsaw puzzle.
**JigRot** [43]	DA and DG	2021	Self-supervised learning by combining jigsaw and rotation.
**TF** [53]	DG	2017	Low-rank parametrized network.
**SLRC** [38]	DG	2017	Low rank constraint.
**CIDDG** [69]	DG	2018	Conditional invariant deep domain generalization.
**MMD-AAE** [33]	DG	2018	Adversarial auto-encoders.
**D-SAM** [70]	DG	2018	Domain-specific aggregation modules.
**MLDG** [40]	DG	2018	Meta learning approach.
**MetaReg** [39]	DG	2018	Meta learning approach.
**MMLD** [71]	DG	2020	Mixture of Multiple Latent Domains.
**ER** [72]	DG	2020	Domain generalization via entropy regularization.
**DADG** [42]	DG	2021	Discriminative adversarial domain generalization.
**WADG** [35]	DG	2021	Wasserstein adversarial domain generalization.

**Table 3:** Multi-source domain adaptation results on PACS (Reset-18). Each column title indicates the name of the domain used as the target. We use bold font to highlight the best results.

PACS-DA		Art_Paint.	Cartoon	Sketches	Photo	Avg.
[64]	Deep All	74.70	72.40	60.10	92.90	75.03
	Dial	87.30	85.50	66.80	97.00	84.15
	DDiscovery	87.70	86.90	69.60	97.00	85.30
[5]	Deep All	77.85	74.86	67.74	95.73	79.05
	JiGen	84.88	81.07	79.05	97.96	85.74
[4]	Deep All	74.70	72.40	60.10	92.90	75.00
	Rot	88.70	86.40	74.90	98.00	87.00
[29]	Deep All	74.70	72.40	60.10	92.90	75.00
	RotC	90.30	87.40	75.10	97.90	87.70
[43]	Deep All	77.83	74.26	65.81	95.71	78.40
	JigRot	89.67	82.87	83.93	**98.17**	88.66
[65]	DANN	82.91	83.83	69.50	96.29	83.13
	DANN (Aug)	89.01	83.06	78.54	97.25	86.96
[65]	CDAN	85.70	88.10	73.10	97.20	86.00
	CDAN+E	87.40	89.40	75.30	97.80	87.50
	CDAN (Aug)	90.67	85.96	80.50	97.43	88.64
	CDAN+E (Aug)	90.28	85.41	81.37	98.08	88.78
[66]	MDD	89.60	88.99	**87.35**	97.78	90.92
	MDD (Aug)	90.28	86.26	85.72	97.54	89.95
	Deep All	77.26	72.64	69.05	95.41	78.59
	Deep All (Aug)	80.03	74.49	67.85	95.27	79.41
	**Ours**	**92.56**	**91.44**	87.08	98.04	**92.28**

**Table 4:** Accuracy (%) on Office-Home for unsupervised domain adaptation (Resnet-50). The bold font highlights the best domain-adaptation results. $A\overset{}{\to }C$ indicates that A (Art) is the source domain and C (Clipart) is the target domain.

Office-Home	$A\overset{}{\to }C$	$A\overset{}{\to }P$	$A\overset{}{\to }R$	$C\overset{}{\to }A$	$C\overset{}{\to }P$	$C\overset{}{\to }R$	$P\overset{}{\to }A$	$P\overset{}{\to }C$	$P\overset{}{\to }R$	$R\overset{}{\to }A$	$R\overset{}{\to }C$	$R\overset{}{\to }P$	Avg.
ResNet-50 [60]	34.9	50.0	58.0	37.4	41.9	46.2	38.5	31.2	60.4	53.9	41.2	59.9	46.1
DAN [1]	43.6	57.0	67.9	45.8	56.5	60.4	44.0	43.6	67.7	63.1	51.5	74.3	56.3
DANN [2]	45.6	59.3	70.1	47.0	58.5	60.9	46.1	43.7	68.5	63.2	51.8	76.8	57.6
JAN [62]	45.9	61.2	68.9	50.4	59.7	61.0	45.8	43.4	70.3	63.9	52.4	76.8	58.3
GPDA [67]	52.9	**73.4**	**77.1**	52.9	66.1	65.6	52.9	44.9	76.1	65.6	49.7	79.2	63.0
CDAN [65]	49.0	69.3	74.5	54.4	66.0	68.4	55.6	48.3	75.9	68.4	55.4	80.5	63.8
Rot [4]	50.4	67.8	74.6	58.7	66.7	67.4	55.7	52.4	77.5	71.0	59.6	81.2	65.3
CDAN+E [65]	50.7	70.6	76.0	57.6	70.0	70.0	57.4	50.9	77.3	70.9	56.7	81.6	65.8
ALDA [28]	53.7	70.1	76.4	60.2	**72.6**	**71.5**	56.8	51.9	77.1	70.2	56.3	82.1	66.6
RotC [29]	51.7	69.0	75.4	60.4	70.3	70.7	57.7	53.3	**78.6**	72.2	59.9	81.7	66.7
**Ours**	**55.1**	69.0	74.5	**62.5**	66.7	69.8	**62.2**	**56.0**	77.7	**73.5**	**61.9**	**82.2**	**67.6**

**Table 5:** Accuracy (%) on ImageCLEF-DA for unsupervised domain adaptation (Resnet-50). The bold font highlights the best domain adaptation results. I→P indicates that ImageNet ILSVRC 2012 is the source domain and Pascal VOC 2012 is the target domain.

ImageCLEF-DA	$I\;\overset{}{\to }\;P$	$P\;\overset{}{\to }\;I$	$I\;\overset{}{\to }\;C$	$C\;\overset{}{\to }\;I$	$C\;\overset{}{\to }\;P$	$P\;\overset{}{\to }\;C$	Avg.
ResNet-50 [60]	74.8	83.9	91.5	78.0	65.5	91.2	80.7
DAN [1]	74.5	82.2	92.8	86.3	69.2	89.8	82.5
Rot [4]	77.9	91.6	95.6	86.9	70.5	94.8	84.2
DANN [2]	75.0	86.0	96.2	87.0	74.3	91.5	85.0
JAN [62]	76.8	88.0	94.7	89.5	74.2	91.7	85.8
CDAN [65]	76.7	90.6	97.0	90.5	74.5	93.5	87.1
MLADA [24]	78.2	91.2	95.5	90.8	76.0	92.2	87.3
CDAN+E [65]	77.7	90.7	**97.7**	91.3	74.2	94.3	87.7
RotC [29]	**78.6**	92.5	96.1	88.9	73.9	**95.9**	87.7
**Ours**	78.1	**92.7**	96.5	**91.6**	**74.9**	**95.9**	**88.2**

**Table 6:** Accuracy (%) on VisDA (Synthetic → Real) for unsupervised domain adaptation (ResNet-50).

Method	JAN [62]	GTA [73]	CDAN [65]	CDAN+E [65]	Ours
Synthetic → Real	61.6	69.5	66.8	70.0	**72.6**

**Table 7:** Domain generalization results on PACS (Alexnet). For details about the meaning of columns and the use of bold fonts, see Table 3.

PACS-DG		Art_Paint.	Cartoon	Sketches	Photo	Avg.
[53]	Deep All	63.30	63.13	54.07	87.70	67.05
	TF	62.86	66.97	57.51	89.50	69.21
[69]	Deep All	57.55	67.04	58.52	77.98	65.27
	DeepC	62.30	69.58	64.45	80.72	69.26
	CIDDG	62.70	69.73	64.45	78.65	68.88
[40]	Deep All	64.91	64.28	53.08	86.67	67.24
	MLDG	66.23	66.88	58.96	88.00	70.01
[70]	Deep All	64.44	72.07	58.07	87.50	70.52
	D-SAM	63.87	70.70	64.66	85.55	71.20
[42]	Deep All	63.12	66.16	60.27	88.65	69.55
	DADG	66.21	70.28	62.18	89.76	72.11
[39]	Deep All	67.21	66.12	55.32	88.47	69.28
	MetaReg	69.82	70.35	59.26	91.07	72.63
[5]	Deep All	66.68	69.41	60.02	89.98	71.52
	JiGen	67.63	71.71	65.18	89.00	73.38
	JiGen (Aug)	71.53	69.50	68.06	91.08	75.04
[43]	Deep All	66.50	69.65	61.42	89.68	71.81
	JigRot	69.70	71.00	66.00	89.60	74.08
[71]	Deep All	68.09	70.23	61.80	88.86	72.25
	MMLD	69.27	72.83	66.44	88.98	74.38
[72]	Deep All	68.35	70.14	90.83	64.98	73.57
	ER	71.34	70.29	89.92	71.15	75.67
[35]	Deep All	63.30	63.13	54.07	87.70	67.05
	WADG	70.21	72.51	70.32	89.81	75.71
	Deep All	68.26	**74.52**	63.65	90.78	74.30
	Deep All (Aug)	73.73	70.09	65.79	92.22	75.45
	**Ours w/o consis.**	73.44	71.42	**73.91**	89.70	77.12
	**Ours**	**74.02**	72.23	72.36	**91.16**	**77.44**

**Table 8:** Domain generalization results on PACS (Resnet-18). For details about the meaning of columns and the use of bold fonts, see Table 3.

PACS-DG		Art_Paint.	Cartoon	Sketches	Photo	Avg.
[42]	Deep All	75.60	72.30	68.10	93.06	77.27
	DADG	79.89	76.25	70.51	94.86	80.38
[70]	Deep All	77.87	75.89	69.27	95.19	79.55
	D-SAM	77.33	72.43	**77.83**	95.30	80.72
[5]	Deep All	77.85	74.86	67.74	95.73	79.05
	JiGen	79.42	75.25	71.35	**96.03**	80.51
	JiGen (Aug)	79.44	71.50	70.86	95.33	79.28
[72]	Deep All	78.93	75.02	96.60	70.48	80.25
	ER	80.70	76.40	96.65	71.77	81.38
[43]	Deep All	77.83	74.26	65.81	95.71	78.40
	JigRot	81.07	73.97	74.67	95.93	81.41
[39]	Deep All	79.90	75.10	69.50	95.20	79.93
	MetaReg	**83.70**	**77.20**	70.30	95.50	81.68
[71]	Deep All	78.34	75.02	65.24	96.21	78.70
	MMLD	81.28	77.16	72.29	96.09	81.83
	Deep All	77.26	72.64	69.05	95.41	78.59
	Deep All (Aug)	80.03	74.49	67.85	95.27	79.41
	**Ours w/o consis.**	81.84	75.05	77.01	95.07	82.24
	**Ours**	82.32	75.70	77.03	95.87	**82.73**

**Table 9:** Domain generalization results on Office-Home (Resnet-18). For details about the meaning of columns and the use of bold fonts, see Table 3.

Office-Home-DG		Art	Clipart	Product	Real-World	Avg.
[70]	Deep All	55.59	42.42	70.34	70.86	59.81
	D-SAM	58.03	44.37	69.22	71.45	60.77
	Deep All	52.15	45.86	70.86	73.15	60.51
[5]	JiGen	53.04	47.51	71.47	72.79	61.20
[35]	WADG	55.34	44.82	72.03	73.55	61.44
[43]	JigRot	58.33	49.67	72.97	75.27	64.06
[42]	Deep All	54.31	41.41	70.31	73.03	59.77
	DADG	55.57	48.71	70.90	73.70	62.22
	Deep All	57.16	49.06	72.22	73.59	63.01
	**Ours**	**59.20**	**54.67**	**73.21**	**73.93**	**65.25**

**Table 10:** Domain generalization results on VLCS (Alexnet). For details about the meaning of columns and the use of bold fonts, see Table 3.

VLCS-DG		Caltech	Labelme	Pascal	Sun	Avg.
[69]	Deep All	85.73	61.28	62.71	59.33	67.26
	DeepC	87.47	62.60	63.97	61.51	68.89
	CIDDG	88.83	63.06	64.38	62.10	69.59
[68]	Deep All	86.10	55.60	59.10	54.60	63.85
	CCSA	92.30	62.10	67.10	59.10	70.15
[38]	Deep All	86.67	58.20	59.10	57.86	65.46
	SLRC	92.76	62.34	65.25	63.54	70.97
[53]	Deep All	93.40	62.11	68.41	64.16	72.02
	TF	93.63	63.49	69.99	61.32	72.11
[33]	MMD-AAE	94.40	62.60	67.70	64.40	72.28
[70]	Deep All	94.95	57.45	66.06	65.87	71.08
	D-SAM	91.75	56.95	58.59	60.84	67.03
[43]	Deep All	96.15	59.05	70.84	63.92	72.49
	JigRot	96.30	59.20	70.73	66.37	73.15
[5]	Deep All	96.93	59.18	71.96	62.57	72.66
	JiGen	96.93	60.90	70.62	64.30	73.19
[71]	Deep All	95.89	57.88	72.01	67.76	73.39
	MMLD	96.66	58.77	71.96	68.13	73.88
[72]	Deep All	97.15	58.07	73.11	68.79	74.28
	ER	96.92	58.26	**73.24**	**69.10**	74.38
[35]	Deep All	92.86	63.10	68.67	64.11	72.19
	WADG	96.68	**64.26**	71.47	66.62	74.76
[42]	Deep All	94.44	61.30	68.11	63.58	71.86
	DADG	96.80	63.44	70.77	66.81	74.76
	Deep All	97.72	63.03	71.93	66.70	74.85
	**Ours**	**98.74**	62.27	72.79	68.16	**75.49**

**Table 11:** Ablation studies of domain adaptation on PACS. The first three columns indicate the types of image transformations applied. Each column title in the middle indicates the name of the domain used as the target. We use bold font to highlight the best results.

PACS-DA							
**Rnd-Color**	**Rnd-Geo**	**Adv-Stn**	**Art_Paint.**	**Cartoon**	**Sketches**	**Photo**	**Avg.**
*√*			93.02	91.51	86.62	98.00	**92.29**
	*√*		91.83	89.45	83.42	97.98	90.67
		*√*	91.85	**91.61**	82.45	97.92	90.96
*√*	*√*		**93.10**	91.01	86.33	**98.14**	92.15
*√*		*√*	92.56	91.44	**87.08**	98.04	**92.28**

**Table 12:** Ablation studies of domain generalization on PACS. The first three columns indicate the types of image transformations applied. Each column title in the middle indicates the name of the domain used as target. We use bold font to highlight the best results.

PACS-DA							
**Rnd-Color**	**Rnd-Geo**	**Adv-Stn**	**Art_Paint.**	**Cartoon**	**Sketches**	**Photo**	**Avg.**
*√*			71.40	72.43	**71.44**	90.20	76.37
	*√*		71.08	72.40	66.98	91.36	75.46
		*√*	70.92	**73.46**	69.66	90.78	76.21
*√*	*√*		73.05	72.15	69.08	**91.64**	76.48
*√*		*√*	**74.02**	72.23	**72.36**	91.16	**77.44**

## Data Availability

The data used to support the study are publicly available. See Section 4.1 for details.

## References

[1] Long M., Cao Y., Wang J., Jordan M.I. Learning Transferable Features with Deep Adaptation Networks. Proceedings of the 32nd International Conference on International Conference on Machine Learning.

[2] Ganin Y., Ustinova E., Ajakan H., Germain P., Larochelle H., Laviolette F., Marchand M., Lempitsky V. (2017). Domain-Adversarial Training of Neural Networks. Domain Adaptation in Computer Vision Applications.

[3] Hoffman J., Tzeng E., Park T., Zhu J.Y., Isola P., Saenko K., Efros A.A., Darrell T. CyCADA: Cycle Consistent Adversarial Domain Adaptation. Proceedings of the 35th International Conference on Machine Learning.

[4] Xu J., Xiao L., Lopez A.M. (2019). Self-Supervised Domain Adaptation for Computer Vision Tasks. IEEE Access.

[5] Carlucci F.M., D’Innocente A., Bucci S., Caputo B., Tommasi T. Domain Generalization by Solving Jigsaw Puzzles. Proceedings of the 2019 IEEE/CVF Conference on Computer Vision and Pattern Recognition (CVPR).

[6] Ranaldi L., Pucci G. (2023). Knowing Knowledge: Epistemological Study of Knowledge in Transformers. Appl. Sci..

[7] Zhang X., Wang Q., Zhang J., Zhong Z. Adversarial AutoAugment. Proceedings of the 8th International Conference on Learning Representations.

[8] Volpi R., Murino V. Addressing Model Vulnerability to Distributional Shifts Over Image Transformation Sets. Proceedings of the 2019 IEEE/CVF International Conference on Computer Vision (ICCV).

[9] Cubuk E.D., Zoph B., Mane D., Vasudevan V., Le Q.V. Autoaugment: Learning augmentation policies from data. Proceedings of the 2019 IEEE/CVF Conference on Computer Vision and Pattern Recognition (CVPR).

[10] Lim S., Kim I., Kim T., Kim C., Kim S. Fast AutoAugment. Proceedings of the Advances in Neural Information Processing Systems.

[11] Cubuk E.D., Zoph B., Shlens J., Le Q.V. RandAugment: Practical data augmentation with no separate search. Proceedings of the Advances in Neural Information Processing Systems.

[12] Sajjadi M., Javanmardi M., Tasdizen T. Regularization with Stochastic Transformations and Perturbations for Deep Semi-Supervised Learning. Proceedings of the Advances in Neural Information Processing Systems.

[13] Xie Q., Dai Z., Hovy E., Luong T., Le Q. Unsupervised Data Augmentation for Consistency Training. Proceedings of the Advances in Neural Information Processing Systems.

[14] Suzuki T., Sato I. Adversarial Transformations for Semi-Supervised Learning. Proceedings of the Thirty-Fourth AAAI Conference on Artificial Intelligence (AAAI-20).

[15] Miyato T., Maeda S.I., Koyama M., Ishii S. (2019). Virtual Adversarial Training: A Regularization Method for Supervised and Semi-Supervised Learning. IEEE Trans. Pattern Anal. Mach. Intell..

[16] Jaderberg M., Simonyan K., Zisserman A., Kavukcuoglu K. Spatial transformer networks. Proceedings of the Advances in Neural Information Processing Systems.

[17] Hendrycks D., Dietterich T. Benchmarking neural network robustness to common corruptions and perturbations. Proceedings of the 7th International Conference on Learning Representations.

[18] Tzeng E., Hoffman J., Zhang N., Saenko K., Darrell T. Deep Domain Confusion: Maximizing for Domain Invariance. Proceedings of the 2014 IEEE Conference on Computer Vision and Pattern Recognition (CVPR).

[19] Zhao F., Liu W., Wen C. (2022). A New Method of Image Classification Based on Domain Adaptation. Sensors.

[20] Sun B., Feng J., Saenko K. Return of frustratingly easy domain adaptation. Proceedings of the Thirtieth AAAI Conference on Artificial Intelligence (AAAI-16).

[21] Sun B., Saenko K. Deep CORAL: Correlation alignment for deep domain adaptation. Proceedings of the European Conference on Computer Vision.

[22] Sun H., Chen X., Wang L., Liang D., Liu N., Zhou H. (2020). C2DAN: An Improved Deep Adaptation Network with Domain Confusion and Classifier Adaptation. Sensors.

[23] Tzeng E., Hoffman J., Saenko K., Darrell T. Adversarial Discriminative Domain Adaptation. Proceedings of the 2017 IEEE Conference on Computer Vision and Pattern Recognition (CVPR).

[24] Fang Y., Xiao Z., Zhang W. (2021). Multi-layer adversarial domain adaptation with feature joint distribution constraint. Neurocomputing.

[25] Zhu J., Park T., Isola P., Efros A.A. Unpaired Image-to-Image Translation using Cycle-Consistent Adversarial Networks. Proceedings of the 2017 IEEE International Conference on Computer Vision (ICCV).

[26] Shu R., Bui H.H., Narui H., Ermon S. A DIRT-T Approach to Unsupervised Domain Adaptation. Proceedings of the 6th International Conference on Learning Representations.

[27] Lee S., Kim D., Kim N., Jeong S.G. Drop to Adapt: Learning Discriminative Features for Unsupervised Domain Adaptation. Proceedings of the 2019 IEEE/CVF International Conference on Computer Vision (ICCV).

[28] Chen M., Zhao S., Liu H., Cai D. Adversarial-Learned Loss for Domain Adaptation. Proceedings of the AAAI Conference on Artificial Intelligence.

[29] Xiao L., Xu J., Zhao D., Wang Z., Wang L., Nie Y., Dai B. Self-Supervised Domain Adaptation with Consistency Training. Proceedings of the 25th International Conference on Pattern Recognition.

[30] Zhao X., Stanislawski R., Gardoni P., Sulowicz M., Glowacz A., Krolczyk G., Li Z. (2022). Adaptive Contrastive Learning with Label Consistency for Source Data Free Unsupervised Domain Adaptation. Sensors.

[31] Muandet K., Balduzzi D., Schölkopf B. Domain Generalization via Invariant Feature Representation. Proceedings of the 30th International Conference on International Conference on Machine Learning.

[32] Ghifary M., Kleijn W.B., Zhang M., Balduzzi D. Domain generalization for object recognition with multi-task autoencoders. Proceedings of the 2015 IEEE International Conference on Computer Vision (ICCV).

[33] Li H., Pan S.J., Wang S., Kot A.C. Domain generalization with adversarial feature learning. Proceedings of the 2018 IEEE/CVF Conference on Computer Vision and Pattern Recognition (CVPR).

[34] Rahman M.M., Fookes C., Baktashmotlagh M., Sridharan S. (2019). Correlation-aware Adversarial Domain Adaptation and Generalization. Pattern Recognit..

[35] Zhou F., Jiang Z., Shui C., Wang B., Chaib-draa B. (2021). Domain generalization via optimal transport with metric similarity learning. Neurocomputing.

[36] Xu Z., Li W., Niu L., Xu D. Exploiting Low-Rank Structure from Latent Domains for Domain Generalization. Proceedings of the European Conference on Computer Vision.

[37] Li W., Xu Z., Xu D., Dai D., Gool L.V. (2017). Domain Generalization and Adaptation using Low Rank Exemplar SVMs. IEEE Trans. Pattern Anal. Mach. Intell..

[38] Ding Z., Fu Y. (2018). Deep Domain Generalization With Structured Low-Rank Constraint. IEEE Trans. Image Process..

[39] Balaji Y., Sankaranarayanan S., Chellappa R. Metareg: Towards domain generalization using meta-regularization. Proceedings of the Advances in Neural Information Processing Systems.

[40] Li D., Yang Y., Song Y.Z., Hospedales T.M. Learning to generalize: Meta-learning for domain generalization. Proceedings of the AAAI Conference on Artificial Intelligence.

[41] Dou Q., Castro D.C., Kamnitsas K., Glocker B. Domain Generalization via Model-Agnostic Learning of Semantic Features. Proceedings of the 33rd International Conference on Neural Information Processing Systems.

[42] Chen K., Zhuang D., Chang J.M. (2022). Discriminative adversarial domain generalization with meta-learning based cross-domain validation. Neurocomputing.

[43] Bucci S., D’Innocente A., Liao Y., Carlucci F.M., Caputo B., Tommasi T. (2021). Self-Supervised Learning Across Domains. IEEE Trans. Pattern Anal. Mach. Intell..

[44] Dosovitskiy A., Springenberg J.T., Riedmiller M., Brox T. Discriminative unsupervised feature learning with convolutional neural networks. Proceedings of the Advances in Neural Information Processing Systems.

[45] DeVries T., Taylor G.W. (2017). Improved Regularization of Convolutional Neural Networks with Cutout. arXiv.

[46] Zhang H., Cisse M., Dauphin Y.N., Lopez-Paz D. mixup: Beyond Empirical Risk Minimization. Proceedings of the 6th International Conference on Learning Representations.

[47] Hendrycks D., Mu N., Cubuk E.D., Zoph B., Gilmer J., Lakshminarayanan B. AugMix: A Simple Data Processing Method to Improve Robustness and Uncertainty. Proceedings of the 8th International Conference on Learning Representations.

[48] Ho D., Liang E., Stoica I., Abbeel P., Chen X. Population Based Augmentation: Efficient Learning of Augmentation Policy Schedules. Proceedings of the 36th International Conference on Machine Learning.

[49] Volpi R., Namkoong H., Sener O., Duchi J.C., Murino V., Savarese S. Generalizing to unseen domains via adversarial data augmentation. Proceedings of the Advances in Neural Information Processing Systems.

[50] Chen W., Tian L., Fan L., Wang Y. Augmentation Invariant Training. Proceedings of the International Conference on Computer Vision Workshop (ICCVW).

[51] Berthelot D., Carlini N., Goodfellow I., Papernot N., Oliver A., Raffel C.A. MixMatch: A Holistic Approach to Semi-Supervised Learning. Proceedings of the Advances in Neural Information Processing Systems.

[52] Grandvalet Y., Bengio Y. Semi-supervised learning by entropy minimization. Proceedings of the Advances in Neural Information Processing Systems.

[53] Li D., Yang Y., Song Y.Z., Hospedales T.M. Deeper, broader and artier domain generalization. Proceedings of the 2017 IEEE International Conference on Computer Vision (ICCV).

[54] Saenko K., Hulis B., Fritz M., Darrel T. Adapting visual category models to new domains. Proceedings of the European Conference on Computer Vision.

[55] Venkateswara H., Eusebio J., Chakraborty S., Panchanathan S. Deep Hashing Network for Unsupervised Domain Adaptation. Proceedings of the 2017 IEEE Conference on Computer Vision and Pattern Recognition (CVPR).

[56] Torralba A., Efros A.A. Unbiased look at dataset bias. Proceedings of the IEEE Conference on Computer Vision and Pattern Recognition.

[57] Recht B., Roelofs R., Schmidt L., Shankar V. (2018). Do cifar-10 classifiers generalize to cifar-10?. arXiv.

[58] Krizhevsky A., Hinton G. (2009). Learning Multiple Layers of Features from Tiny Images.

[59] Krizhevsky A., Sutskever I., Hinton G.E. ImageNet Classification with Deep Convolutional Neural Networks. Proceedings of the 25th International Conference on Neural Information Processing Systems.

[60] He K., Zhang X., Ren S., Sun J. Deep Residual Learning for Image Recognition. Proceedings of the 2016 IEEE Conference on Computer Vision and Pattern Recognition (CVPR).

[61] Deng J., Dong W., Socher R., Li L.J., Li K., Fei-Fei L. ImageNet: A Large-Scale Hierarchical Image Database. Proceedings of the 2009 IEEE Conference on Computer Vision and Pattern Recognition.

[62] Long M., Zhu H., Wang J., Jordan M.I. Deep Transfer Learning with Joint Adaptation Networks. Proceedings of the 34th International Conference on Machine Learning.

[63] Carlucci F.M., Porzi L., Caputo B., Ricci E., Bulo S.R. Just dial: Domain alignment layers for unsupervised domain adaptation. Proceedings of the International Conference on Image Analysis and Processing.

[64] Mancini M., Porzi L., RotaBulo S., Caputo B., Ricci E. Boosting domain adaptation by discovering latent domains. Proceedings of the 2018 IEEE/CVF Conference on Computer Vision and Pattern Recognition (CVPR).

[65] Long M., Cao Z., Wang J., Jordan M.I. Conditional Adversarial Domain Adaptation. Proceedings of the Advances in Neural Information Processing Systems.

[66] Zhang Y., Liu T., Long M., Jordan M. Bridging Theory and Algorithm for Domain Adaptation. Proceedings of the 36th International Conference on Machine Learning.

[67] Sun J., Wang Z., Wang W., Li H., Sun F. (2021). Domain adaptation with geometrical preservation and distribution alignment. Neurocomputing.

[68] Motiian S., Piccirilli M., Adjeroh D.A., Doretto G. Unified deep supervised domain adaptation and generalization. Proceedings of the 2017 IEEE International Conference on Computer Vision (ICCV).

[69] Li Y., Tian X., Gong M., Liu Y., Liu T., Zhang K., Tao D. Deep domain generalization via conditional invariant adversarial networks. Proceedings of the European Conference on Computer Vision.

[70] D’Innocente A., Caputo B. Domain generalization with domain-specific aggregation modules. Proceedings of the 40th German Conference on Pattern Recognition (GCPR).

[71] Matsuura T., Harada T. Domain Generalization Using a Mixture of Multiple Latent Domains. Proceedings of the AAAI Conference on Artificial Intelligence.

[72] Zhao S., Gong M., Liu T., Fu H., Tao D., Larochelle H., Ranzato M., Hadsell R., Balcan M.F., Lin H. (2020). Domain Generalization via Entropy Regularization. Proceedings of the Advances in Neural Information Processing Systems.

[73] Sankaranarayanan S., Balaji Y., Castillo C.D., Chellappa R. Generate to Adapt: Aligning Domains Using Generative Adversarial Networks. Proceedings of the 2018 IEEE/CVF Conference on Computer Vision and Pattern Recognition.

[74] Sun Y., Wang X., Liu Z., Miller J., Efros A.A., Hardt M. Test-Time Training for Out-of-Distribution Generalization. Proceedings of the 37th International Conference on Machine Learning.

[75] van der Maaten L., Hinton G. (2008). Visualizing Data using t-SNE. J. Mach. Learn. Res..

